# Supplementary material for: Endothelial tip-cell position, filopodia formation and biomechanics require BMPR2 expression and signaling
Source: Commun Biol. 2025 Jan 8;8:21. doi: 10.1038/s42003-024-07431-8 (PMC11711618; doi:10.1038/s42003-024-07431-8)
Supplement: Supplementary file 1 — Supplementary Information [file 42003_2024_7431_MOESM1_ESM.pdf]

Supplementary information

Endothelial tip-cell position, filopodia formation and biomechanics require BMPR2 expression and signaling

Christian Hiepen[1†*], Mounir Benamar[1*], Jorge Barrasa-Fano[2], Mar Condor[2], Mustafa Ilhan[1,3], Juliane Münch[4], Nurcan Hastar[1], Yannic Kerkhoff[1], Gregory S. Harms[5], Thorsten Mielke[6], Benjamin Koenig[7], Stephan Block[1], Oliver Rocks[8], Salim Abdelilah-Seyfried[4], Hans Van Oosterwyck[2,9], Petra Knaus[1,7]

[1]Freie Universitaet Berlin, Institute for Chemistry and Biochemistry, Thielallee 63, 14195 Berlin, Germany

[†]Current affiliation: Westphalian University of Applied Sciences, August-Schmidt-Ring 10, 45665 Recklinghausen, Germany

[2]KU Leuven, Department of Mechanical Engineering, Biomechanics section, Leuven, Celestijnenlaan 300C, 3001 Leuven, Belgium

[3]Berlin School of Integrative Oncology, Augustenburger Platz 1, D-13353 Berlin, Germany

[4]Universität Potsdam, Institute of Biochemistry and Biology, Karl-Liebknecht Strasse 24-25, 14476 Potsdam-Golm, Germany

[5]Universitaetsmedizin, Johannes Gutenberg-Universität Mainz, Cell Biology Unit, Imaging Core Facility and the Research Center for Immune Intervention, Langenbeckstraße 1, 55131 Mainz, Germany

[6]Max-Planck-Institute for Molecular Genetics, Microscopy & Cryo-Electron Microscopy, Ihnestraße 63-73, 14195 Berlin, Germany

[7]Leibniz Forschungsinstitut für Molekulare Pharmakologie (FMP), Robert-Rössle-Straße 10, 13125 Berlin, Germany

[8]Charité - Universitätsmedizin Berlin, Systemic Cell Dynamics, Charitéplatz 1, 10117 Berlin, Germany

[9]KU Leuven, Prometheus Division of Skeletal Tissue Engineering, Herestraat 49, 3000 Leuven, Belgium

This file includes:

Supplementary Figures 1-25

*These authors contributed equally: Christian Hiepen, Mounir Benamar

Corresponding authors: Petra Knaus & Christian Hiepen

Petra.knaus@fu-berlin.de; christian.hiepen@w-hs.de

Supplementary Figure 1

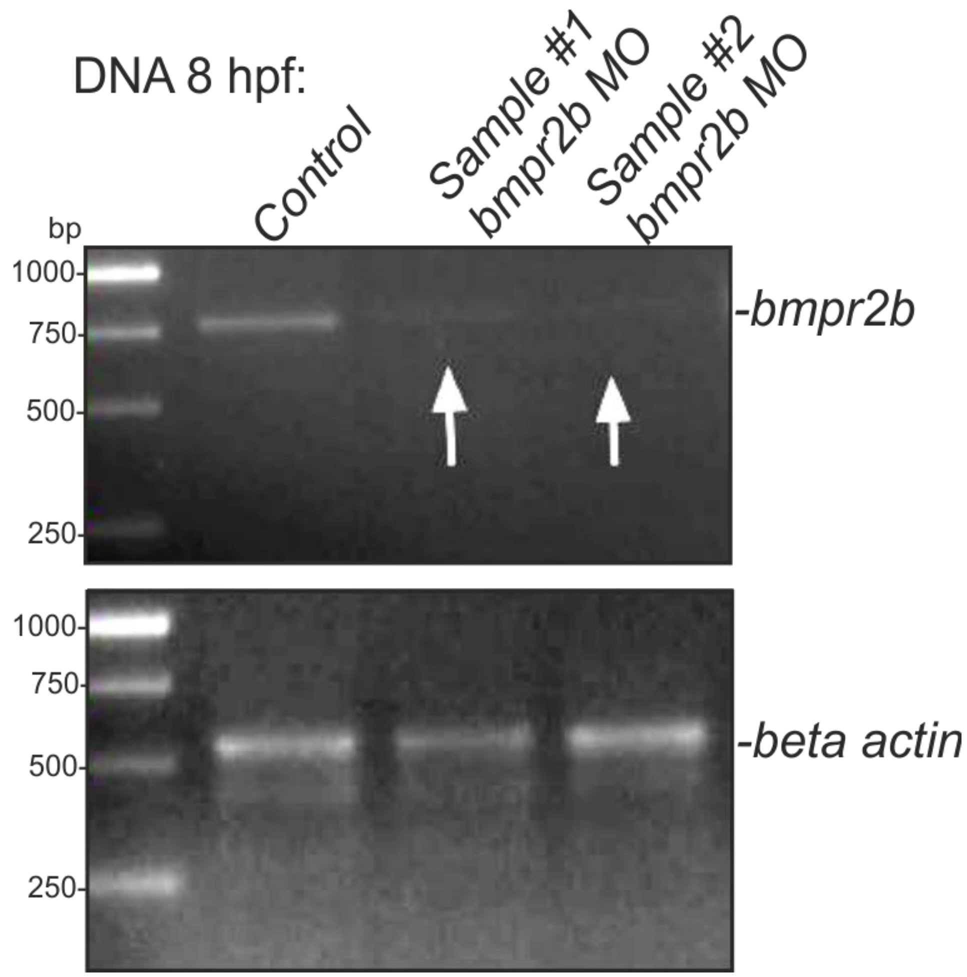

Supplementary Figure 1: *bmpr2b* was targeted in samples consisting of Zebrafish embryos by using a splice morpholino and its expression was analyzed by semiquantitative results of reverse transcription PCR at 8 hrs post fertilization (hpf) relative to beta actin PCR. Arrows indicate loss of *bmpr2b* transcript upon injection of morpholino E2 in two different experiments.

Supplementary Figure 2

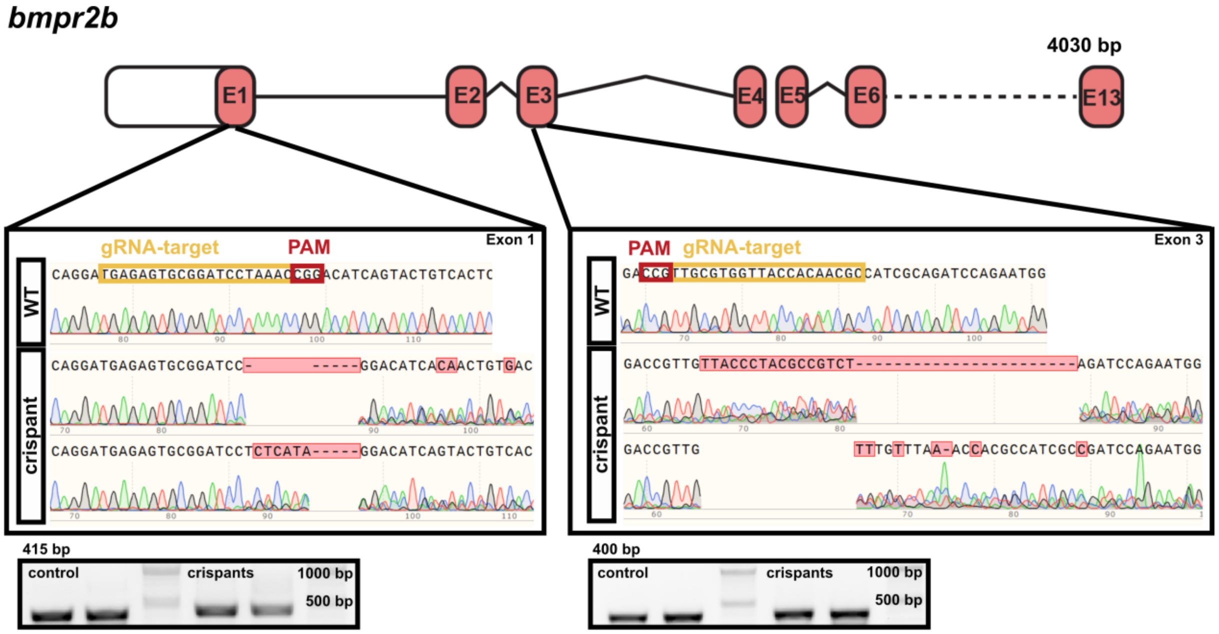

Supplementary Figure 2: Scheme representing the zebrafish bmpr2b gene indicates a part of the sequence of exon 1 and exon 3. Sanger-sequencing of the PCR product (gel pictures in lower panels) revealed clear peaks (wildtype sequence in control embryos) or overlapping peaks adjacent to the protospacer adjacent motif (PAM) in bmpr2b-crispant embryos, indicating successful mosaic mutagenesis. Two representative examples for each mutated exon are shown.

Supplementary Figure 3

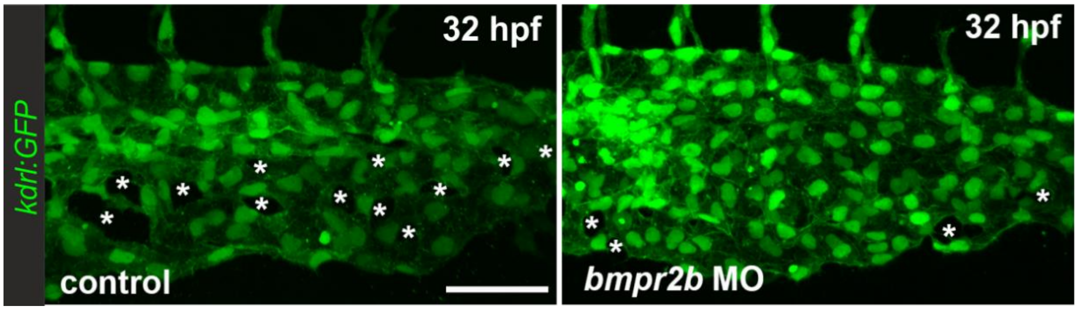

Supplementary Figure 3: Representative images of kdrl-GFP reporter expressing zebrafish treated with control or BMPR2-targeting morpholinos (*bmpr2b* MO) at 32 hrs post fertilization (hpf). Maximum projection images show the caudal vein plexus (CVP) from kdrl:GFP control and bmpr2b MO-treated embryos. Asterisks indicate the fenestrations of the plexus, which are reduced in *bmpr2b* morphant CVP. Scale bar: 50 µm.

Supplementary Figure 4

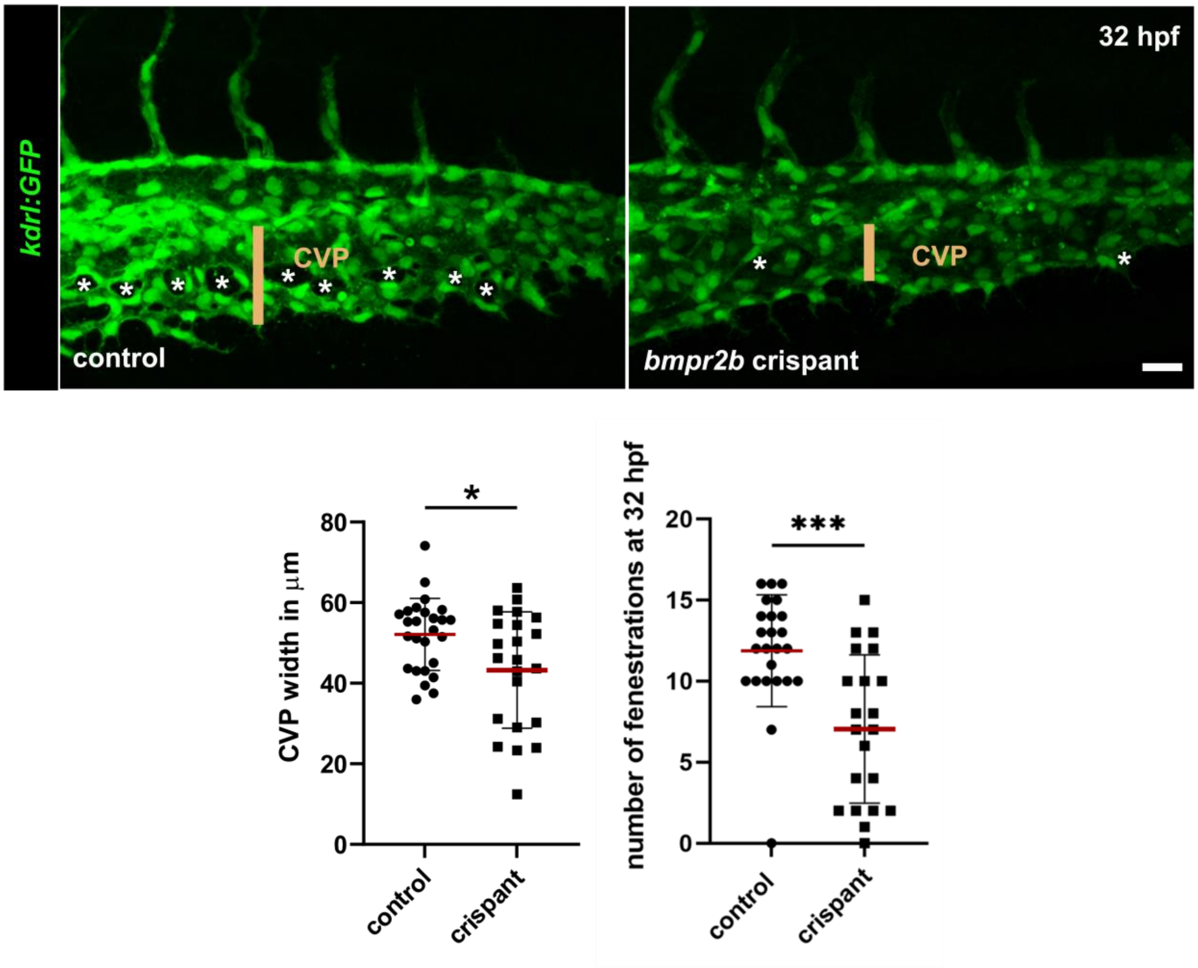

Supplementary Figure 4: Representative images (maximum projection) of the CVP from Tg(kdrl:GFP) control or bmpr2b-crispants at 32 hrs post fertilization (hpf). White asterisks mark fenestrations, a line indicates the size of the CVP. Scale bar: 20 µm. The graph indicates the size of the CVP in control or bmpr2b crispant embryos at 32 hpf. *$p<0.05$. Quantification of fenestrations of the CVP in control or bmpr2b crispant embryos at 32 hpf. ***$p<0.001$. Quantification data are shown as mean ± SD (each point represents independent embryo). Statistical significance was obtained using a two-sample student's t-test.

Supplementary Figure 5

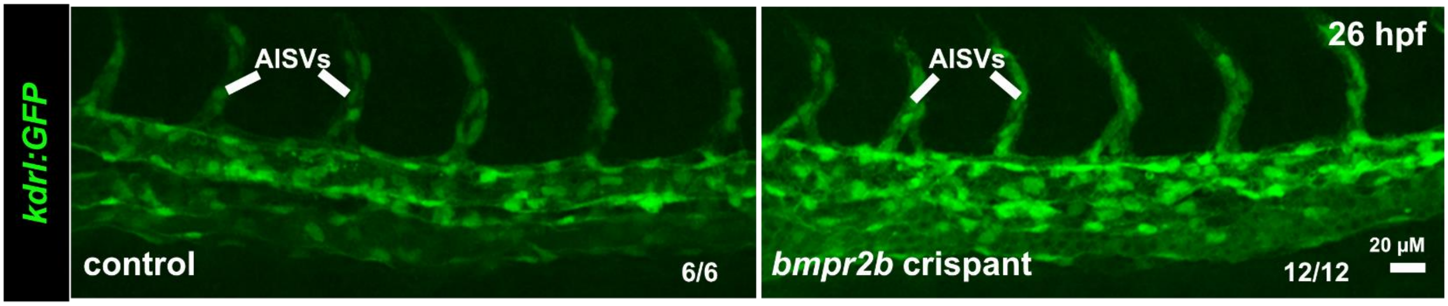

Supplementary Figure 5: Representative maximum projection images of the trunk of Tg(kdrl:GFP) control or bmpr2b-crispants at 32 hpf, showing arterial intersomitic vessels (AISVs). 6 out of 6 or 12 out of 12 replicates show this phenotype. Scale bar: 20 µm

Supplementary Figure 6

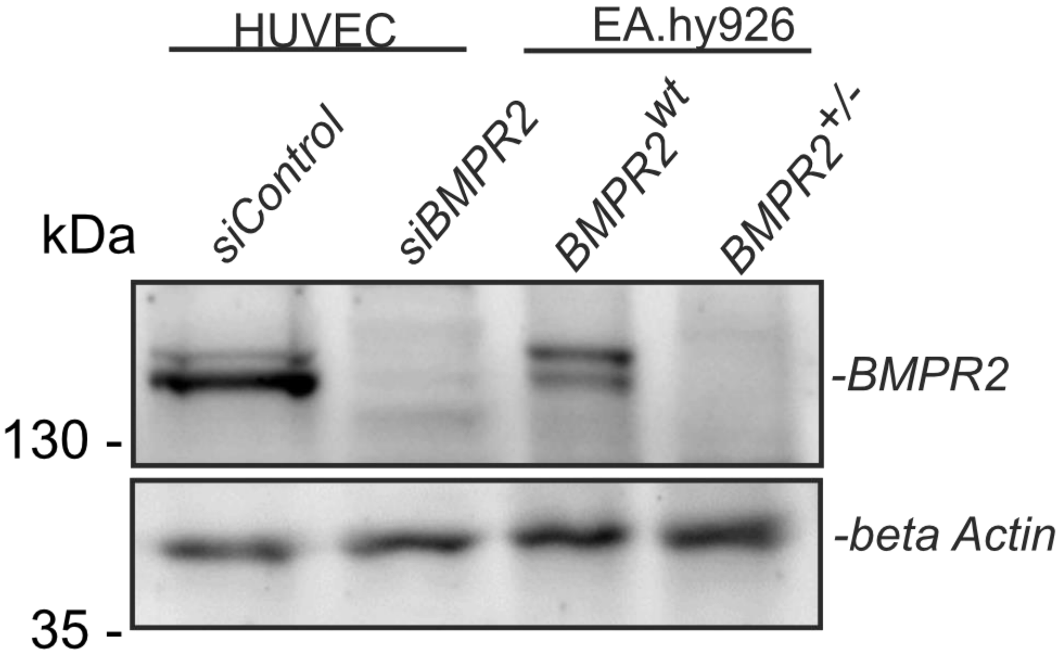

Supplementary Figure 6: Immunoblot against BMPR2 and beta Actin: Cell extracts of HUVECs transfected with siControl or siBMPR2 silencing-RNA and BMPR2 expression in $BMPR2^{wt}$ or $BMPR2^{+/-}$ CRISPR/Cas9 edited EC cell line EA.hy926 (parental cell type = HUVECs).

Supplementary Figure 7

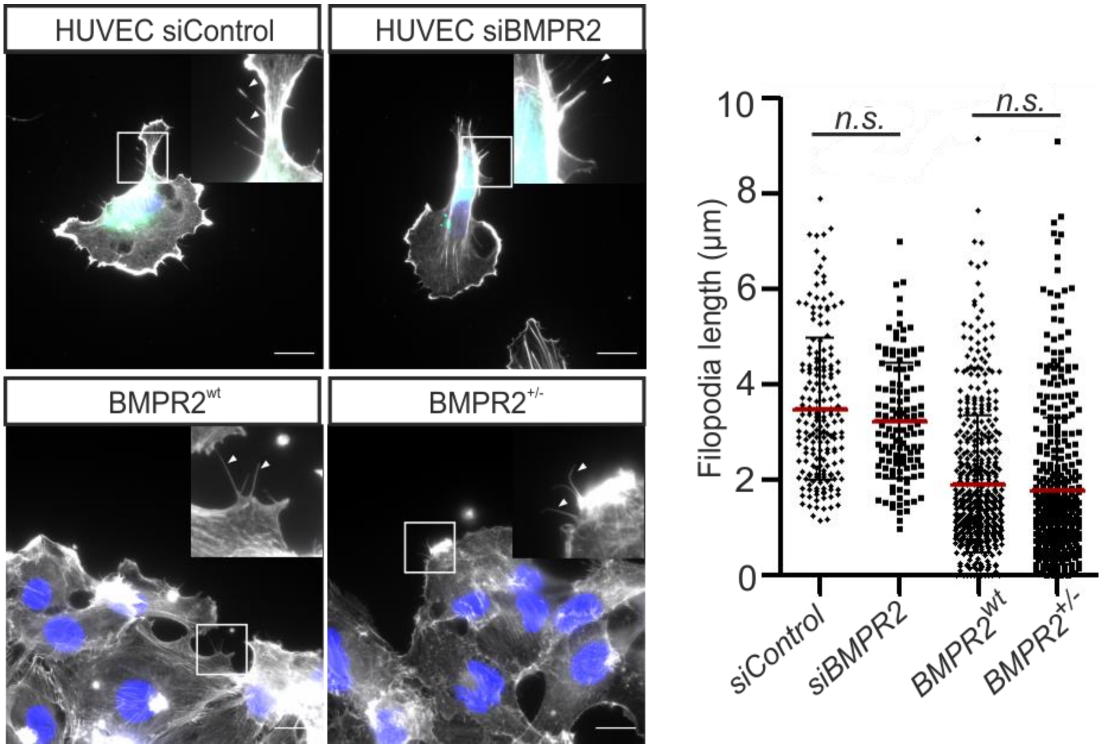

Supplementary Figure 7: Representative images of filopodia and quantification of filopodia length in HUVECs transfected with siControl or siBMPR2 siRNA and in $BMPR2^{wt}$ or $BMPR2^{+/-}$ ECs. Phalloidin (white), DAPI (blue), FITC-labeled siRNA (green). Scale bar: 20µm. n.s.: $p > 0.05$. Quantification data are shown as mean ± SD (n = 3 independent experiments). Statistical significance was obtained using a two-sample student's t-test.

Supplementary Figure 8

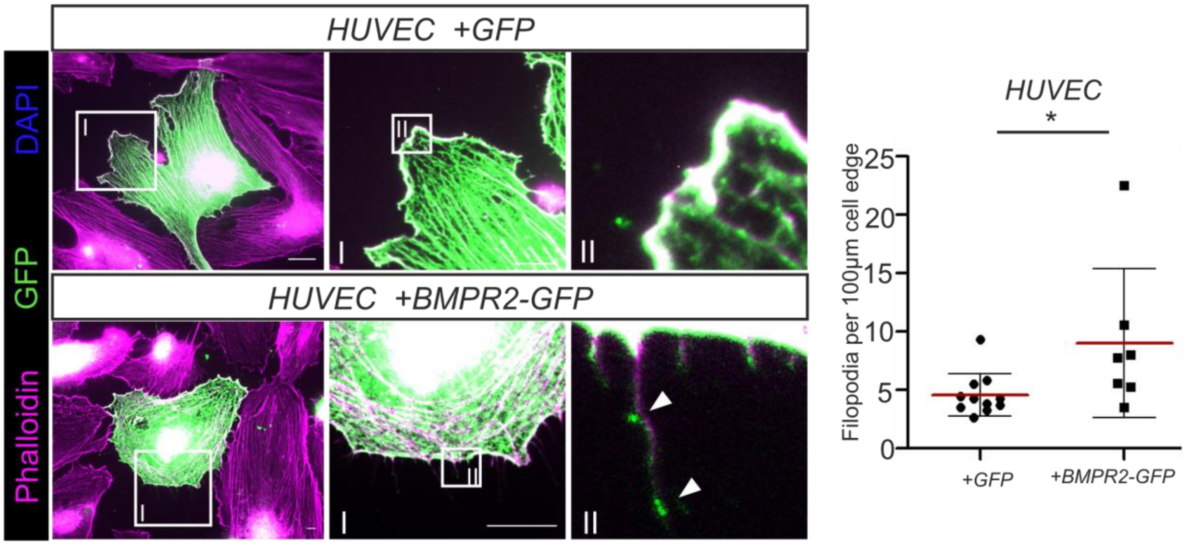

Supplementary Figure 8: Immunofluorescence staining of HUVEC transfected with GFP control or BMPR2-GFP expression plasmids and corresponding quantification of the number of filopodia per 100 µm of cell edge. Phalloidin (magenta), DAPI (blue), anti-GFP antibody (green). Insets (II) show regions of interest. Scale bar 20 µm in main images and 10µm in zoom-in. *$p<0.05$. Quantification data are shown as mean ± SD (n = 3 independent experiments). Statistical significance was obtained using a two-sample student's t-test.

Supplementary Figure 9

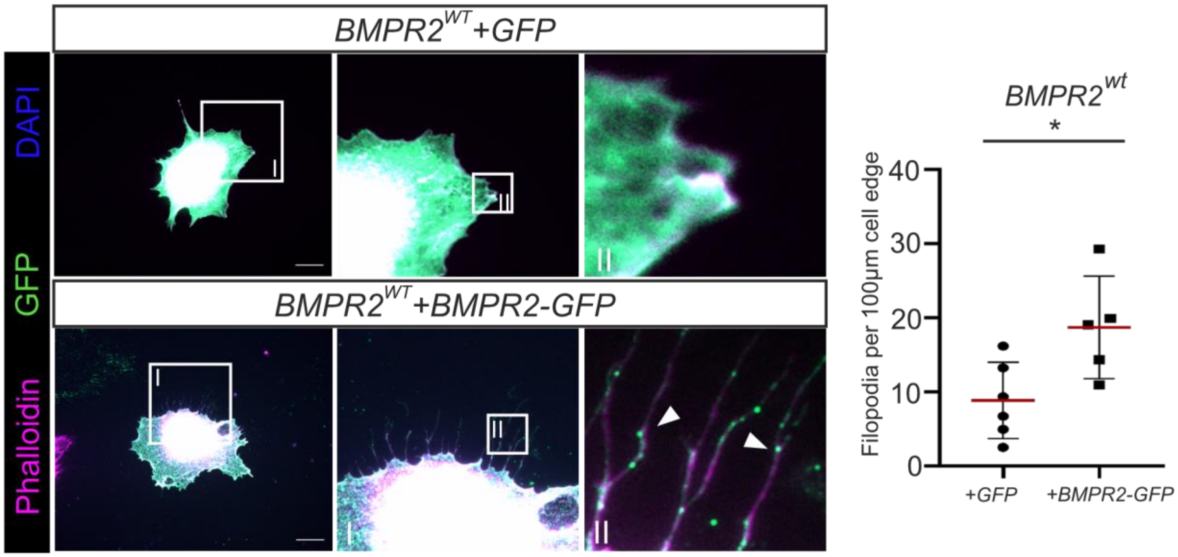

Supplementary Figure 9: Immunofluorescence staining of BMPR2^{wt} ECs transfected with GFP control or BMPR2-GFP expression plasmids and corresponding quantification of the number of filopodia per 100 µm of cell edge. Phalloidin (magenta), DAPI (blue), anti-GFP antibody (green). Insets (II) show regions of interest. Scale bar 20 µm in main images and 10µm in zoom-in. *p<0.05. Quantification data are shown as mean ± SD (n = 3 independent experiments). Statistical significance was obtained using a two-sample student's t-test.

Supplementary Figure 10

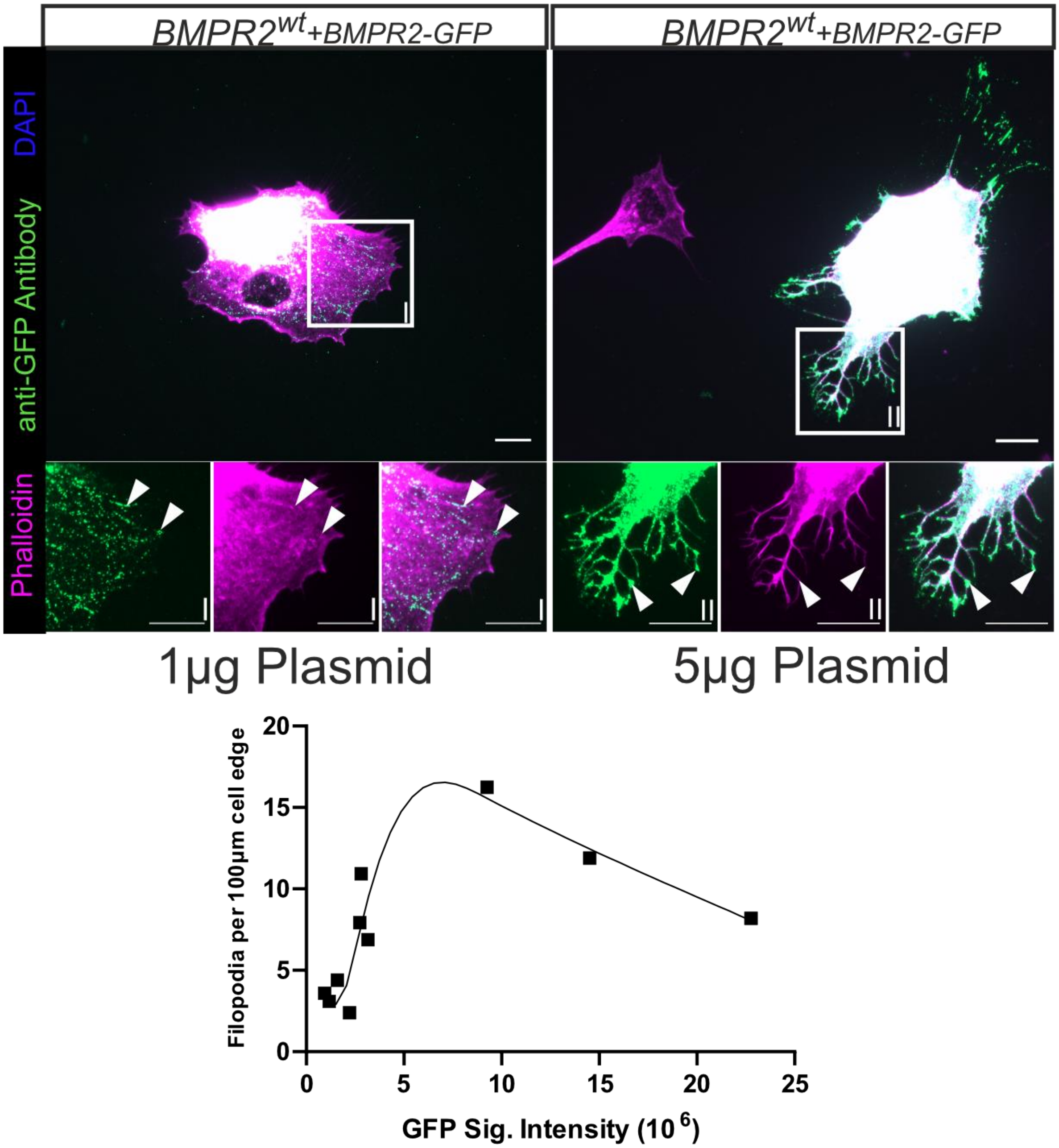

Supplementary Figure 10: Immunofluorescence staining of BMPR2^{wt} ECs co-transfected with different amounts of BMPR2-GFP expression vector (1 µg and 5 µg/ 25.000 cells). Phalloidin (magenta), DAPI (blue), GFP (green). Arrowheads indicate BMPR2 localization hotspots. Insets show regions of interest. Scale bar: 10 µm. Lower graph shows the number of filopodia plotted against the total GFP signal per cell for ECs transfected with 1.4 µg of DNA after seeding in a 12-well plate. Data fitted using LOESS (Locally Estimated Scatterplot Smoothing)

Supplementary Figure 11

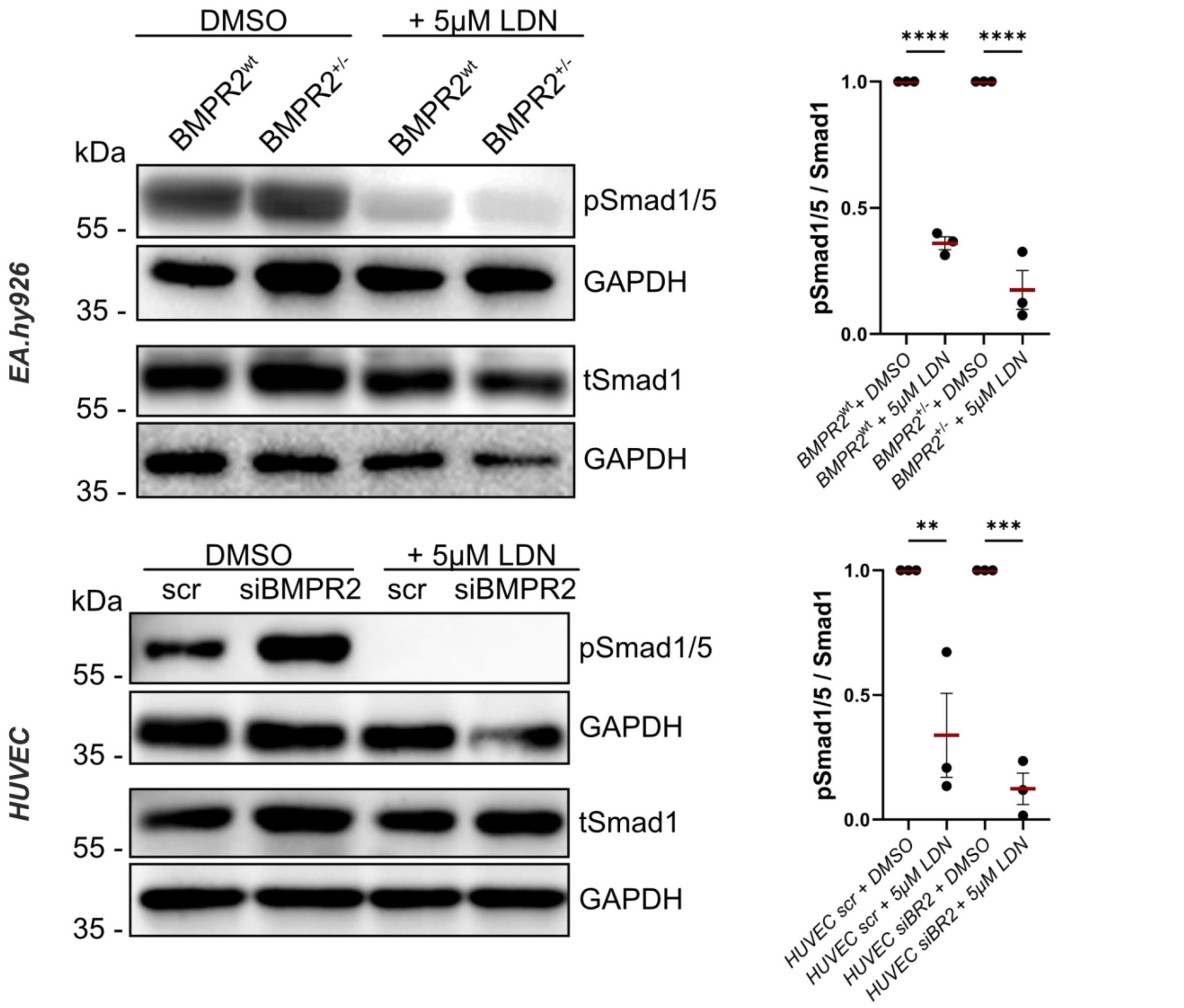

Supplementary Figure 11: Immunoblot against pSmad 1/5/9, total Smad1 (tSmad1) and GAPDH from BMPR2^{wt} and BMPR2$^{+/-}$ ECs and HUVECs treated with scrambled (scr) siRNA or BMPR2-targeting (siBMPR2) siRNA before treatment with DMSO or 5µM of small molecule inhibitor LDN-193189 for 60mn in EC activation medium containing 20 % Serum and angiogenic growth factors. Quantification of the immunoblots indicates fold increase of pSmad1/5 / tSmad1 signal for ECs treated with 5 µM LDN-193189 in comparison to DMSO treatment only. *p<0.05; **p<0.005; ***p<0.001; ****p<0.0001. Quantification data are shown as mean ± SD (n = 3 independent experiments). Statistical significance was obtained using a two-sample student's t-test.

Supplementary Figure 12

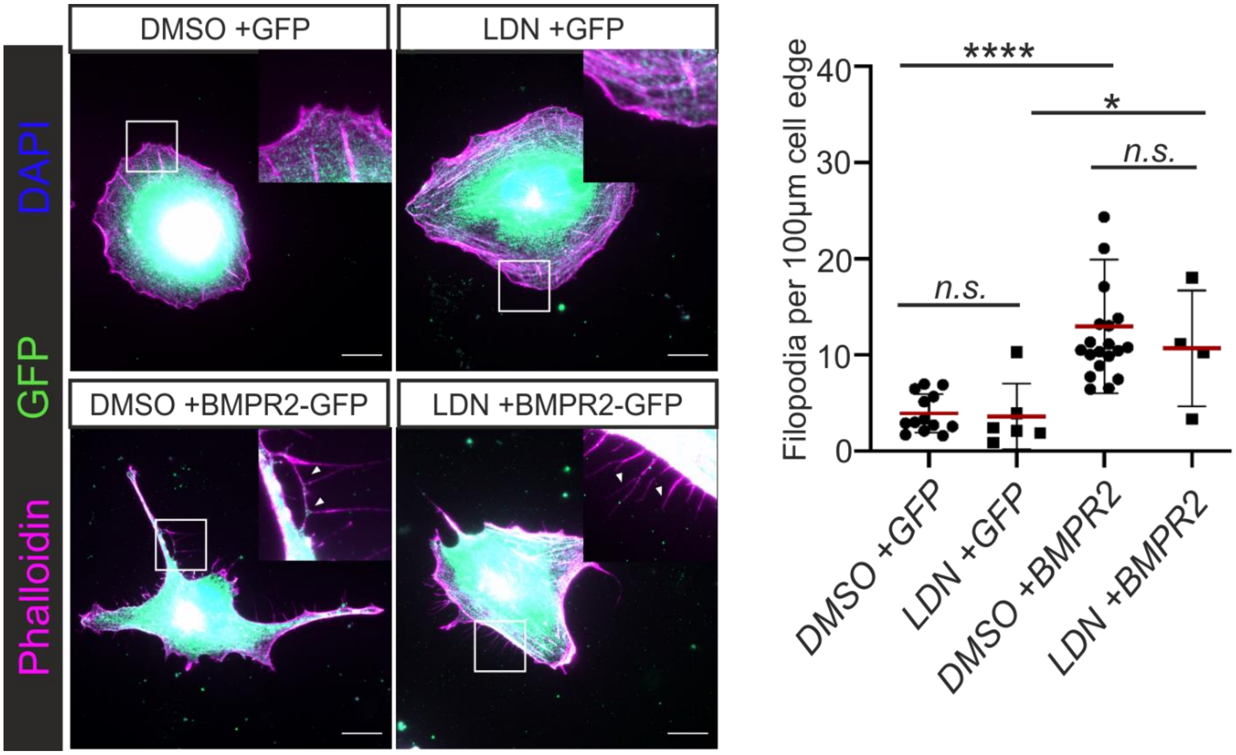

Supplementary Figure 12: Representative images of $BMPR2^{+/-}$ ECs overexpressing GFP control or BMPR2-GFP and treated with DMSO or 5 µM LDN-193189 for 60mn and quantification of the number of filopodia per 100 µm of cell edge for each condition. LDN treatment did not induce significant reduction in number of Filopodia per 100 µm cell edge. Phalloidin (magenta), DAPI (blue), anti-GFP antibody (green). Arrows indicate BMPR2-GFP positive filopodia. Scale bar: 20µm *p<0.05; ****p<0.0001. Quantification data are shown as mean ± SD (n = 3 independent experiments). Statistical significance was obtained using two-sample student's t-tests.

Supplementary Figure 13

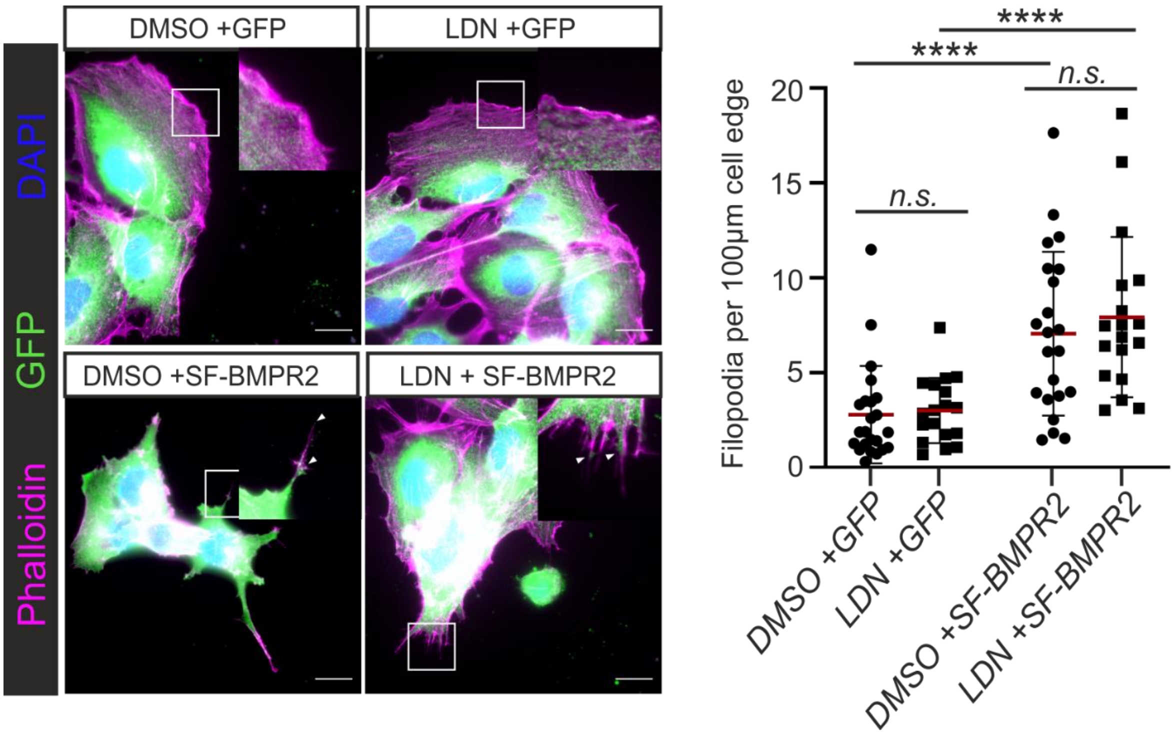

Supplementary Figure 13: Representative images of BMPR2$^{+/-}$ ECs overexpressing GFP control or Short Form (SF)-BMPR2 and treated with DMSO or 5 µM LDN-193189 for 60mn and quantification of the number of filopodia per 100 µm of cell edge for each condition. While SF-BMPR2 overexpression induced increased filopodia formation, LDN treatment did not induce significant reduction in number of Filopodia per 100 µm cell edge. Phalloidin (magenta), DAPI (blue), anti-GFP antibody (green). Arrows indicate BMPR2-GFP positive filopodia. Scale bar: 20µm *$p<0.05$; ****$p<0.0001$. Quantification data are shown as mean ± SD (n = 3 independent experiments). Statistical significance was obtained using two-sample student's t-tests.

Supplementary Figure 14

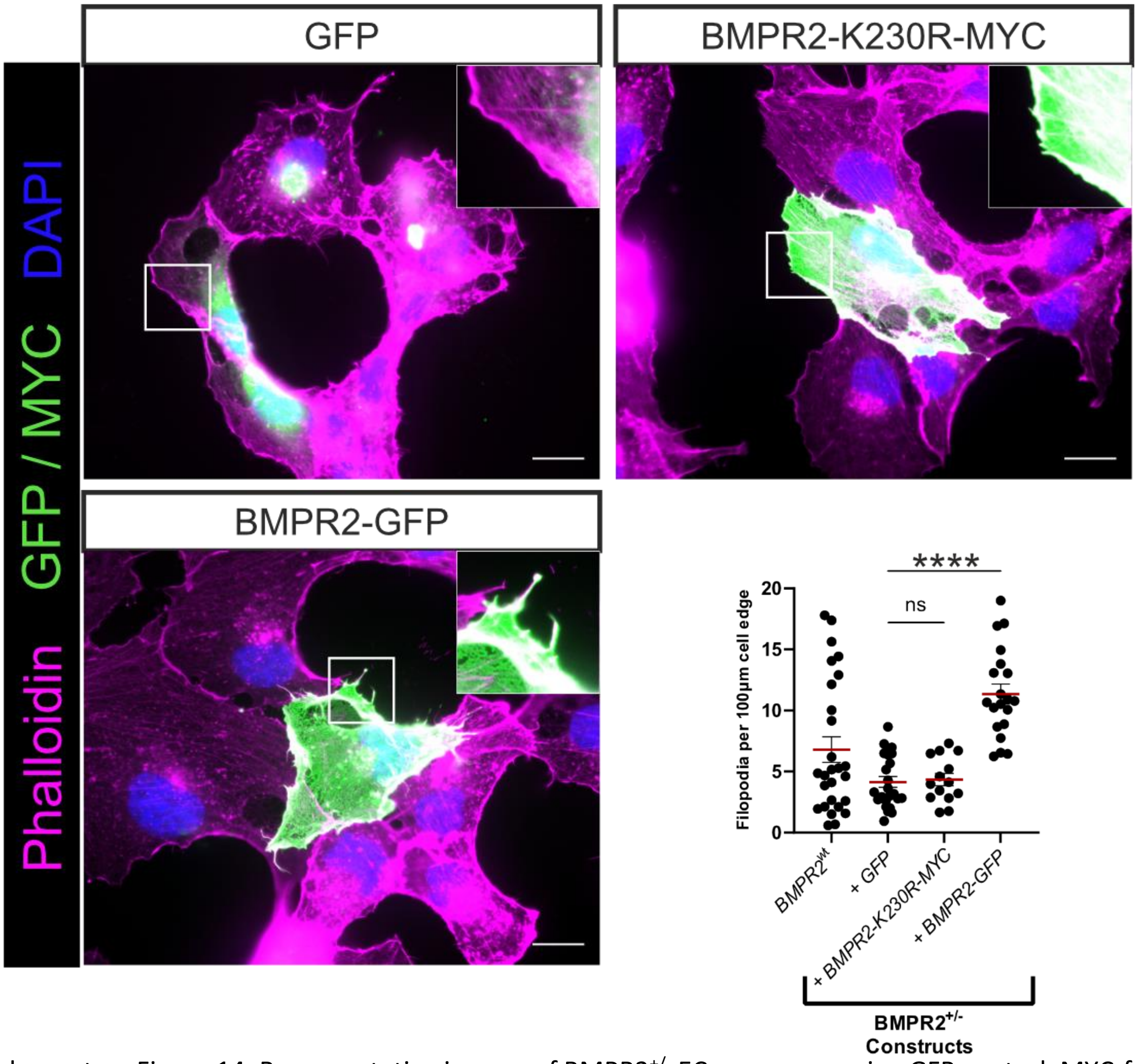

Supplementary Figure 14: Representative images of BMPR2$^{+/-}$ ECs overexpressing GFP control, MYC-fused kinase dead BMPR2 (BMPR2-K230R-MYC) or BMPR2-GFP and quantification of the number of filopodia per 100 µm of cell edge for each condition. While BMPR2-GFP overexpression rescued filopodia formation in BMPR2$^{+/-}$ ECs, overexpression of BMPR2-K230R-MYC did not induce significant increase in number of Filopodia per 100 µm cell edge in comparison to GFP overexpression control. Phalloidin (magenta), DAPI (blue), GFP / anti-MYC antibody (green). Scale bar: 20µm. ns: non-significant; ****$p<0.0001$. Quantification data are shown as mean ± SD (n = 3 independent experiments). Statistical significance relative to GFP -transfected control cells was calculated using one-way ANOVA and Tukey's post-hoc test.

Supplementary Figure 15

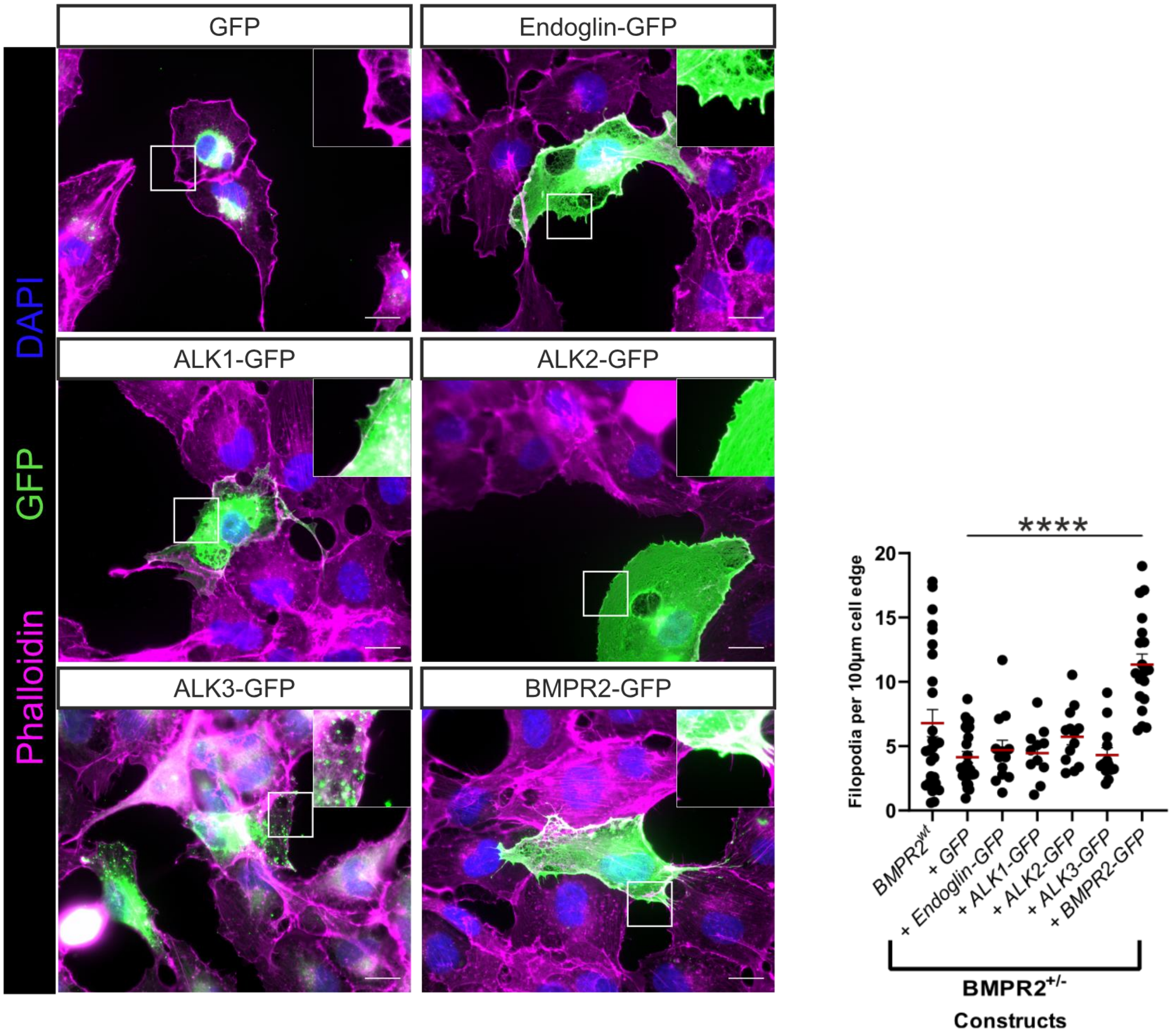

Supplementary Figure 15: Representative images of BMPR2$^{+/-}$ ECs overexpressing GFP control, Endoglin-GFP, ALK1-GFP, ALK2-GFP, ALK3-GFP or BMPR2-GFP and quantification of the number of filopodia per 100 µm of cell edge for each condition. While BMPR2-GFP overexpression rescued filopodia formation in BMPR2$^{+/-}$ ECs, overexpression of type 1 receptors or Endoglin did not induce significant increase in number of Filopodia per 100 µm cell edge in comparison to GFP overexpression control. Phalloidin (magenta), DAPI (blue), GFP (green). Scale bar: 20µm. ****$p<0.0001$. Quantification data are shown as mean ± SD (n = 3 independent experiments). Statistical significance relative to GFP -transfected control cells was calculated using one-way ANOVA and Tukey's post-hoc test.

Supplementary Figure 16

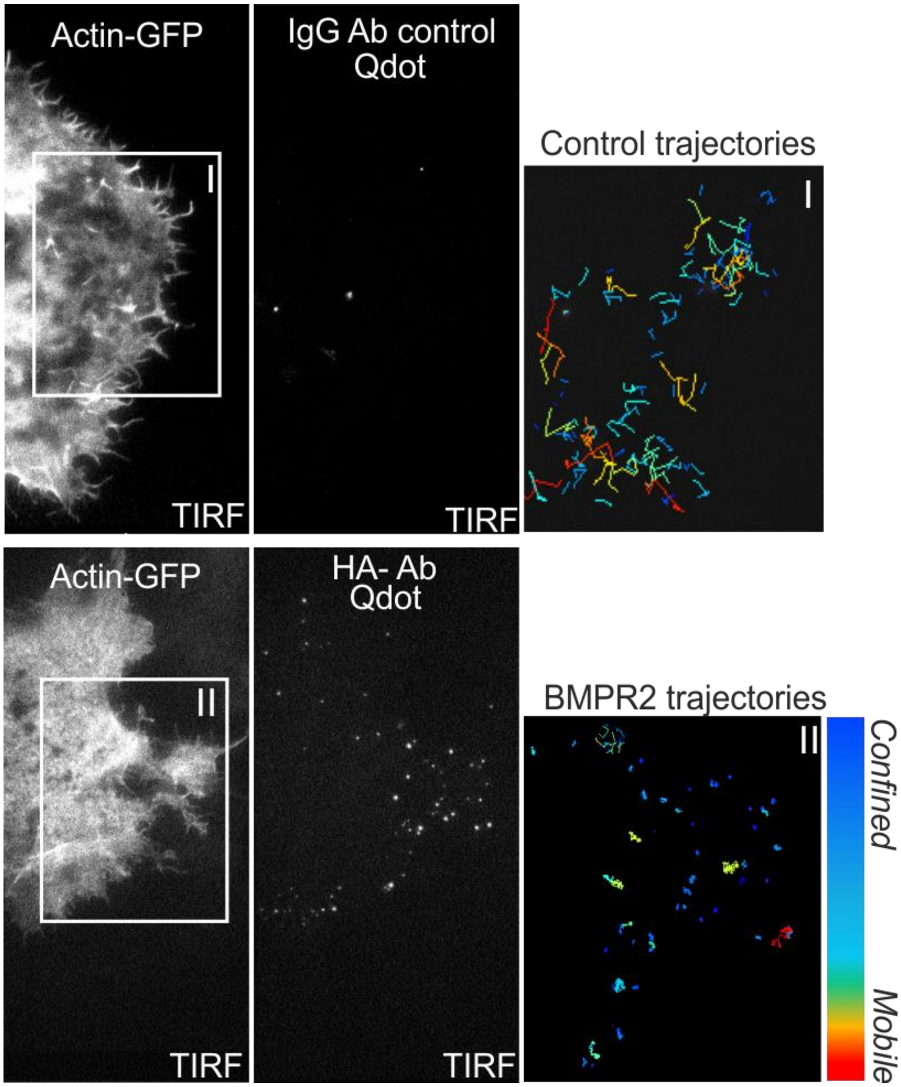

Supplementary Figure 16: Single-Particle Tracking Microscopy (SPTM) of BMPR2-HA overexpressed in Cos7 cells and labeled using control IgG-QDot antibody (top = no binding) or anti-HA-QDot antibody (bottom = BMPR2 binding). Cos7 cells were transfected with LifeAct-GFP prior to imaging and imaged by Total internal reflection fluorescence (TIRF) microscopy. Detected QDot trajectories were overlayed onto the selected cell and color-coded relative to their displacement magnitude. Insets show regions of interest. The control trajectories are much shorter (in terms of number of frames) and have much longer step lengths than the BMPR2 trajectories.

Supplementary Figure 17

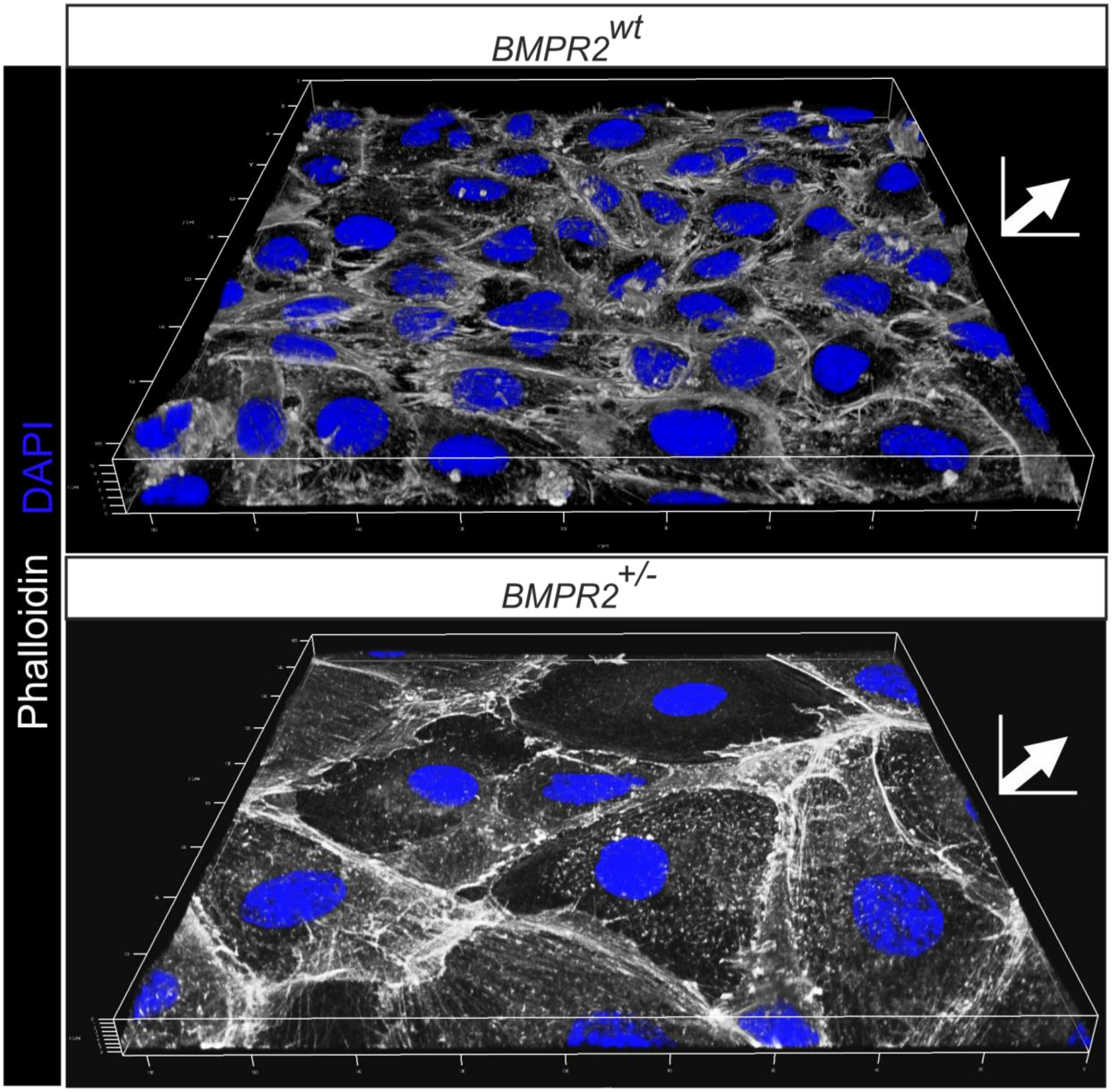

Supplementary Figure 17: 3D reconstruction from confocal z-scan images of BMPR2^{wt} ECs or BMPR2$^{+/-}$ ECs in a gap closure assay (see directionality top right corner). Filamentous actin (F-actin) cytoskeleton was stained by Phalloidin (white), DAPI (blue).

Supplementary Figure 18

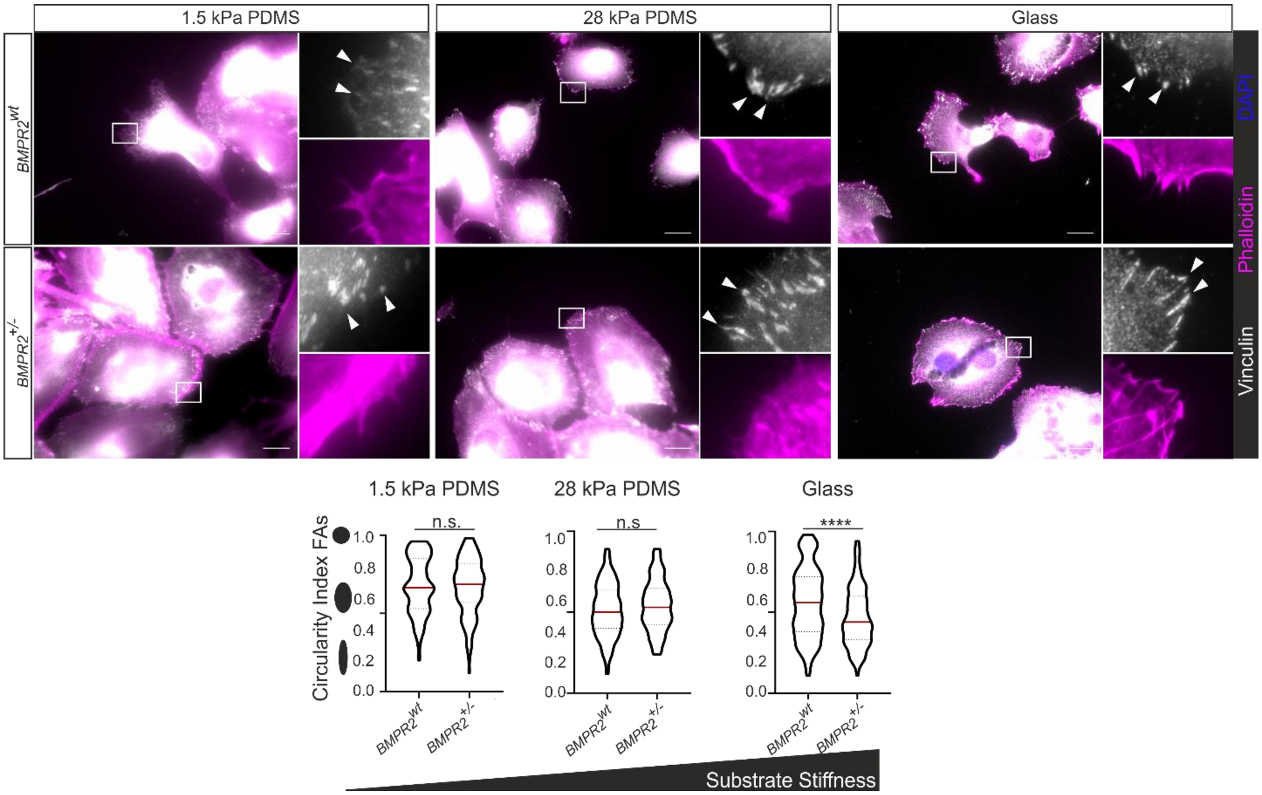

Supplementary Figure 18: Immunofluorescence staining of BMPR2^{wt} ECs and BMPR2$^{+/-}$ ECs seeded on 1.5kPA PDMS, 28 kPa PDMS or fibronectin coated glass. Phalloidin (magenta), DAPI (blue), Vinculin (white) stainings are indicated. Arrows indicate the localization of vinculin-rich focal adhesions (FA), particularly at the cell periphery. Insets show regions of interest. Scale bar: 20 µm. Quantification of FA circularity index (circularity is simplified on the y-axis) for BMPR2^{wt} ECs and BMPR2$^{+/-}$ ECs seeded on 1.5 kPa PDMS substrate, 28 kPa Polydimethylsiloxan (PDMS) substrate (fibronectin fibronectin-coated) or fibronectin fibronectin-coated glass. ****p<0.0001. Quantification data are shown as mean ± SD (n = 3 independent experiments). Statistical significance was obtained using a two-sample student’s t-test.

Supplementary Figure 19

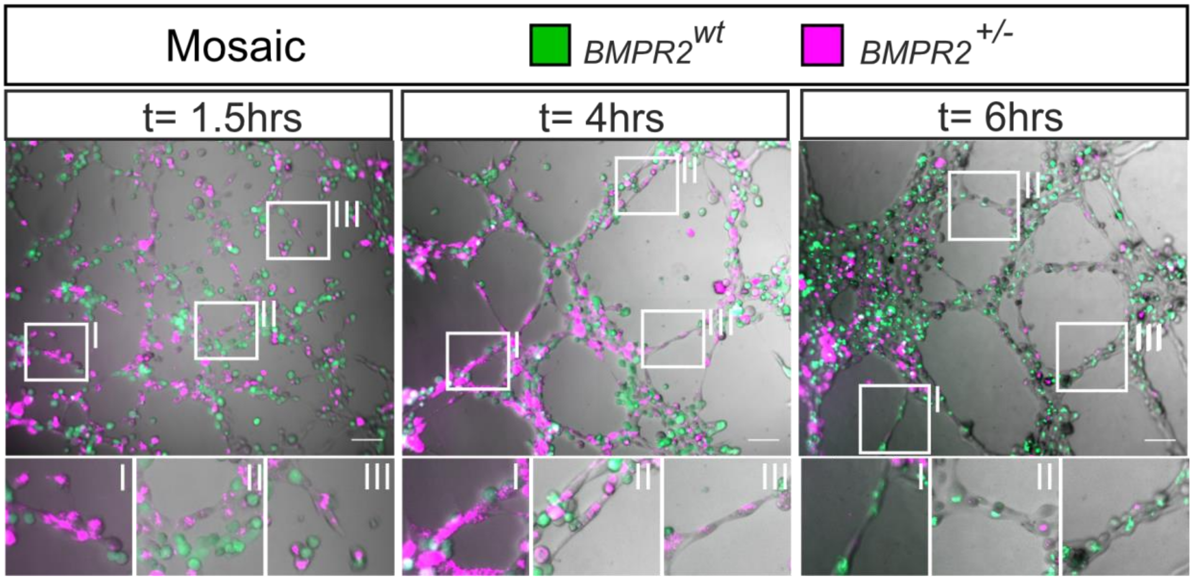

Supplementary Figure 19: Mosaic tube formation assay. Pre-labeled BMPR2^{wt} ECs (green) and BMPR2$^{+/-}$ ECs (magenta) were seeded in Matrigel in a 1:1 ratio and imaged after 1,5 hrs, 4 hrs and 6 hrs upon seeding on soft Matrigel respectively. Insets show tubes that originated by EC adhesion and during tube- fusion. Scale bar: 20µm

Supplementary Figure 20

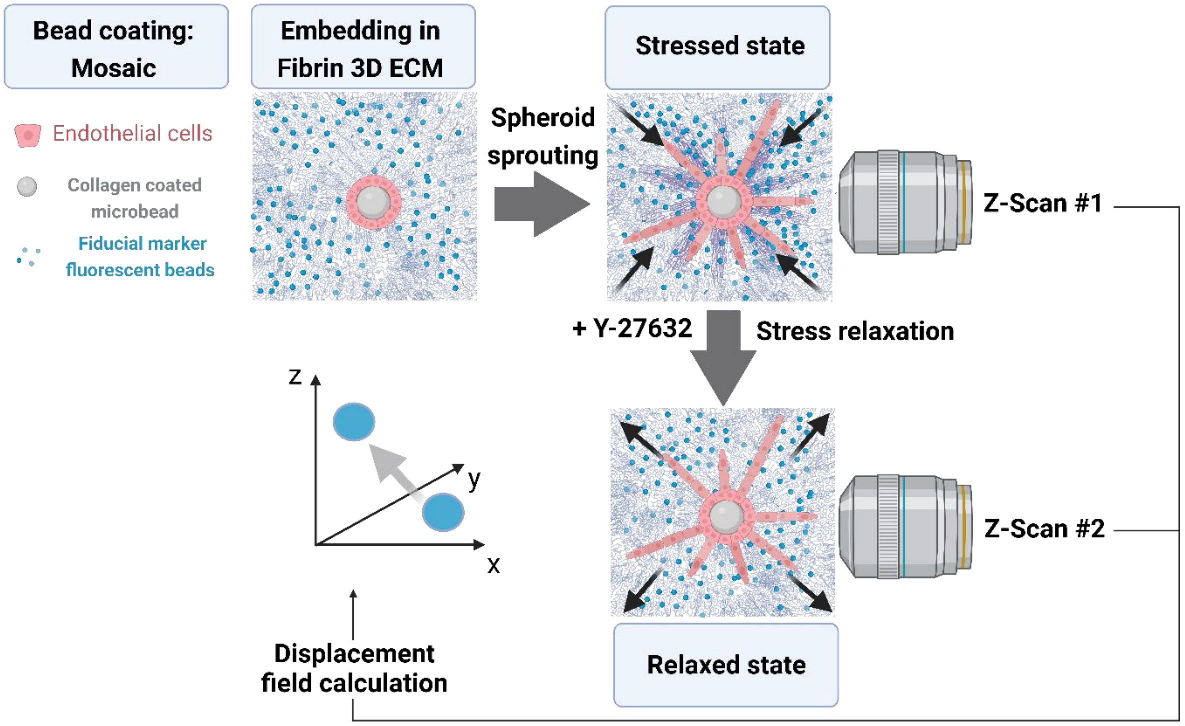

Supplementary Figure 20: Schematic representation of the 3D Traction Force Microscopy (3D TFM) experimental workflow for ECM displacement calculation. Briefly, microcarrier beads (used as fiducial markers to detect vector-based displacements of ECM by EC pulling forces) are coated ECs to obtain fully coated spheroids. EC- coated spheroids are embedded in Fibrin gel polymerizing in-situ together with beads upon addition of thrombin to fibrinogen. Spheroid sprouts form during 64+ hrs, leading to ECM deformations of the Fibrin:marker beads gel by EC interaction with the fibrin ECM via integrin-transmitted pulling forces. After imaging sprouts in their stressed state, cell relaxation is induced experimentally by inhibiting ROCK activity (addition of Y-27632 small molecule inhibitor) required for phosphorylation of Myosin II, a key component of actomyosin contractility. Relaxation of ECs is leading to a relaxed matrix state and consequent ECM/fiducial marker displacement. Displacements are calculated from bead positions in stressed state versus relaxed state through Free Form Deformation (FFD)-based non-rigid image registration of 3D confocal stacks of beads (116).

Supplementary Figure 21

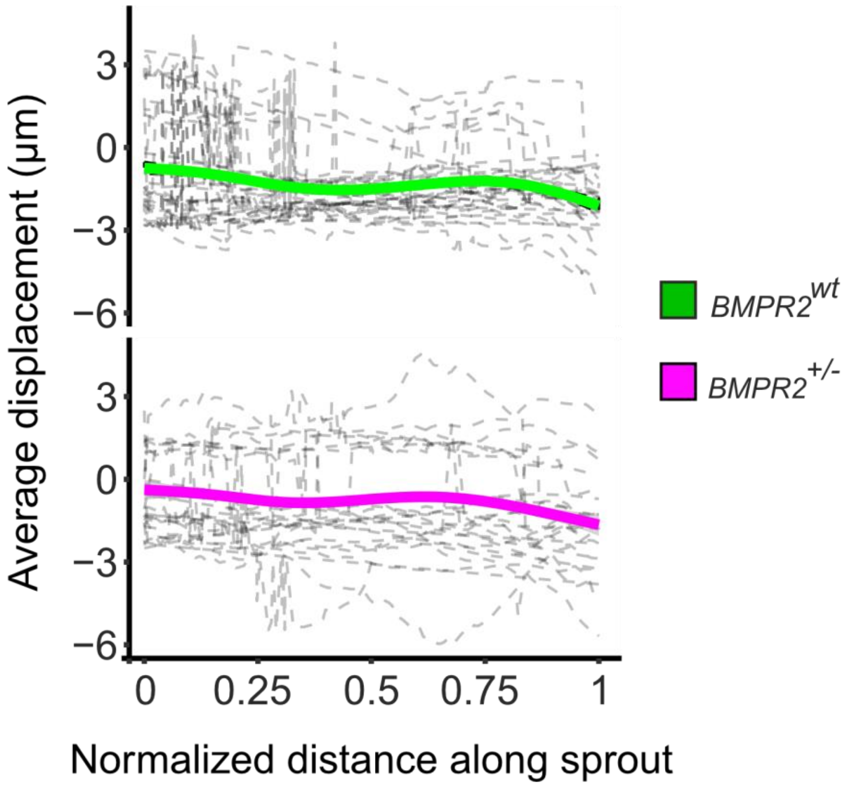

Supplementary Figure 21: Average 3D TFM displacement measured along the normalized distance of sprouts of various lengths for $BMPR2^{wt}$ ECs and $BMPR2^{+/-}$ ECs. Dashed lines represent individual sprout measurements. Colored lines represent averaged displacements for all sprouts per condition.

Supplementary Figure 22

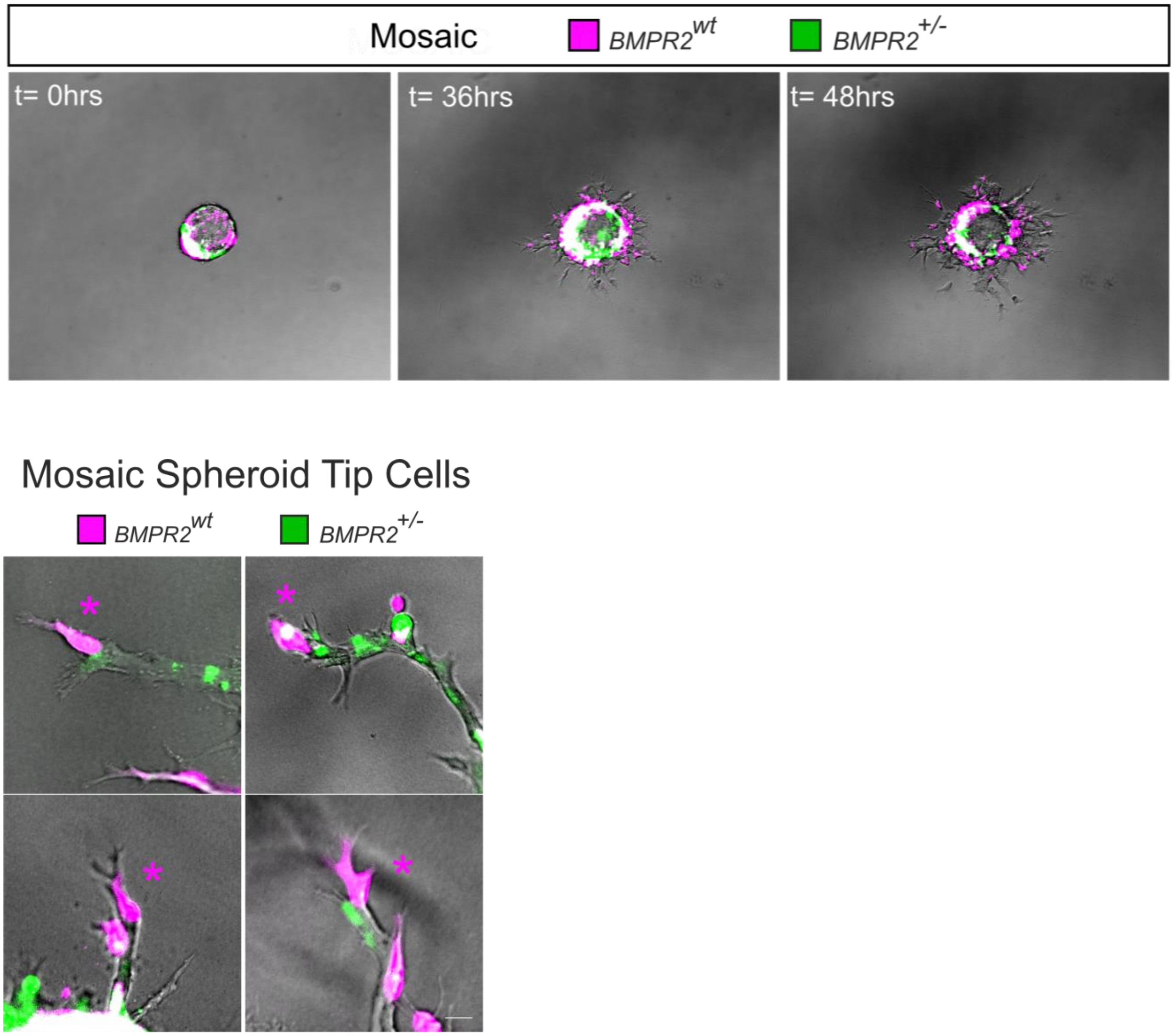

Supplementary Figure 22: Reverse control labelling of MOSAIC spheroids with BMPR2^{wt} ECs (magenta) and BMPR2$^{+/-}$ ECs (green) to rule out dye effects for tip cell position. Cells were seeded in fibrin for sprouting and imaged at 16 hrs, 36 hrs and 48 hrs after embedding. Representative images of tip cells from reverse MOSAIC spheroids after 48hrs of sprouting. In this spheroid labelling approach, BMPR2^{wt} ECs and BMPR2$^{+/-}$ ECs located to their respective position (BMPR2^{wt} cells always at the tip) independent of the dye color used. Magnified region of interest shows sprouts with individual tip cells in green and stalk cells in magenta upon 60 hrs of sprouting. Asterisks indicate position of TC. Scale bar: 40 µm

Supplementary Figure 23

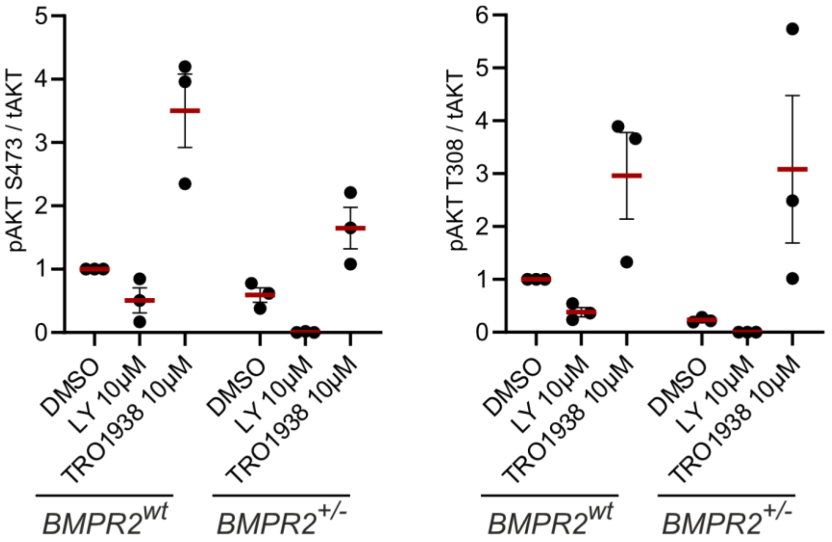

Supplementary Figure 23: Quantification of immunoblots against pAKT-Ser473 and pAKT-Thr308 from BMPR2wt ECs or BMPR2+/- ECs treated with either DMSO, 10µM LY294002 or 10µM UCL-TRO-1938 for 60 min in EC activation medium. pAKT signal was normalized to total AKT for each condition. Quantification data are shown as mean ± SD (n = 3 independent experiments).

Supplementary Figure 24

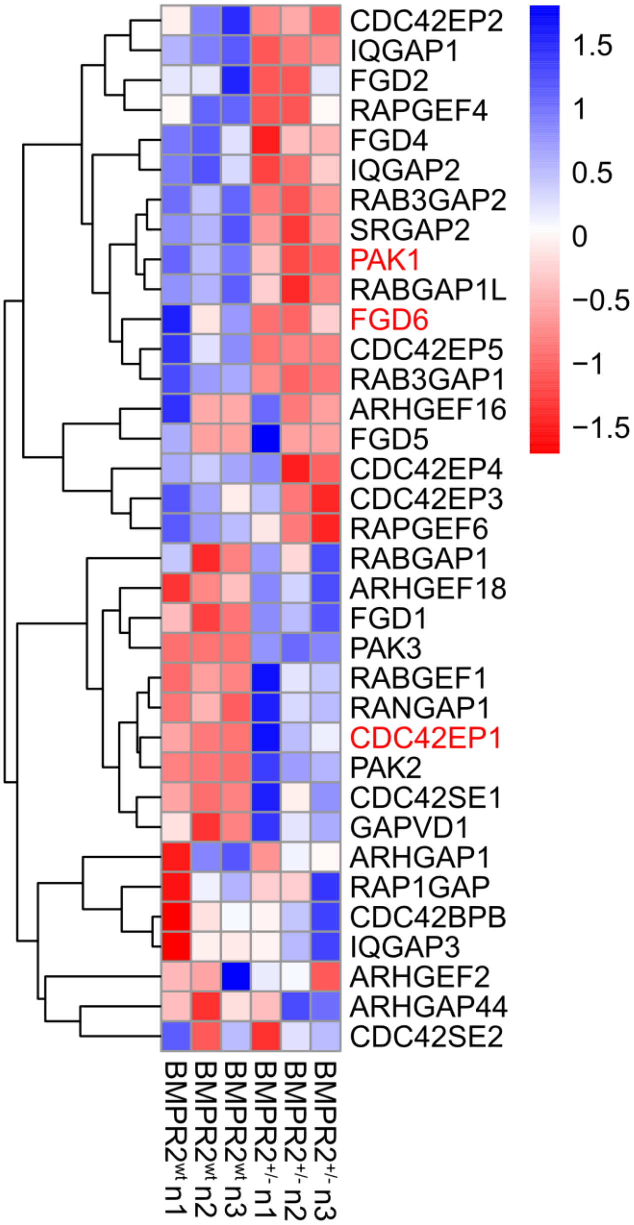

Supplementary Figure 24: Heatmap representation of CDC42-relevant gene expression profiles between $BMPR2^{wt}$ and $BMPR2^{+/-}$ ECs. The figure showcases expression levels of key genes across n=3 experimental repeats for both $BMPR2^{wt}$ and $BMPR2^{+/-}$ ECs. The gradient signifies Z-scores, with red representing lower Z-score values (below the mean) and blue representing higher Z-score values (above the mean). Each row corresponds to a specific gene, while each column represents a different sample. Notably, PAK1, CDC42EP1 (BORG5) and FGD6 are highlighted in red to emphasize their differential expression patterns between $BMPR2^{wt}$ and $BMPR2^{+/-}$ ECs.

Supplementary Figure 25

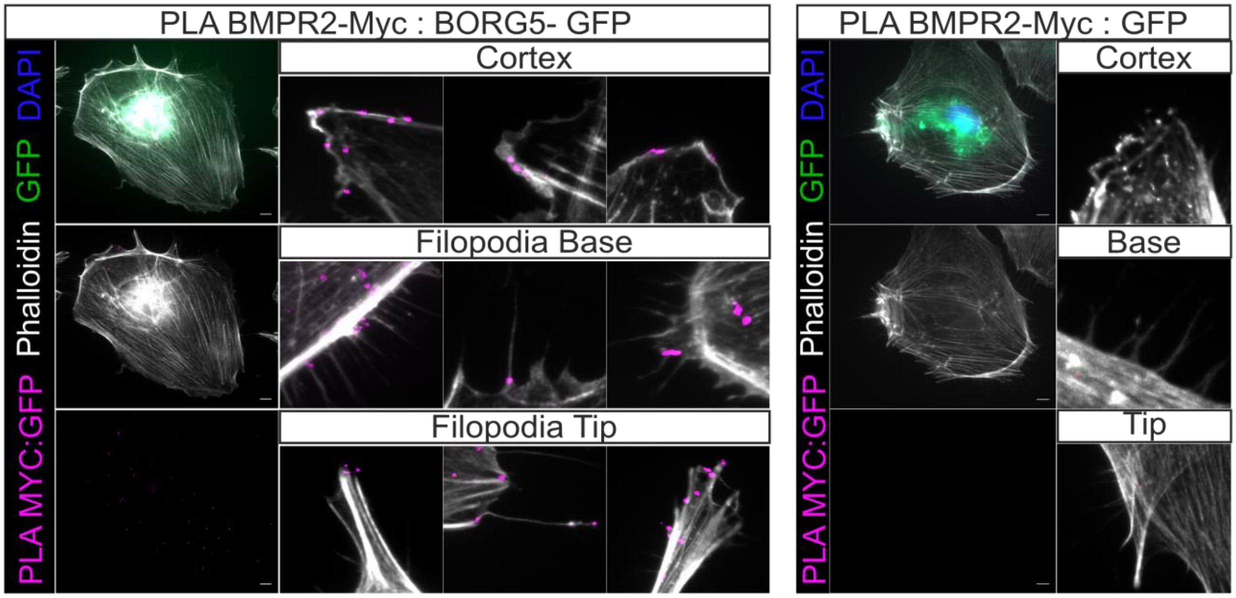

Supplementary Figure 25: Proximity ligation assay (PLA) between Myc and GFP in Cos7 cells overexpressing Myc-tagged BMPR2 (BMPR2-myc) and co-expressing either GFP-tagged BORG5 (BORG5-GFP) or GFP control only. Myc:GFP PLA signal is visible as magenta-coloured single foci at the cortical F-actin rich plasma membrane, the filopodia shaft and tips. Phalloidin (white), DAPI (blue), GFP (green). Insets show regions of interest. Scale bar: 10 µm.

Supplementary Figure 26

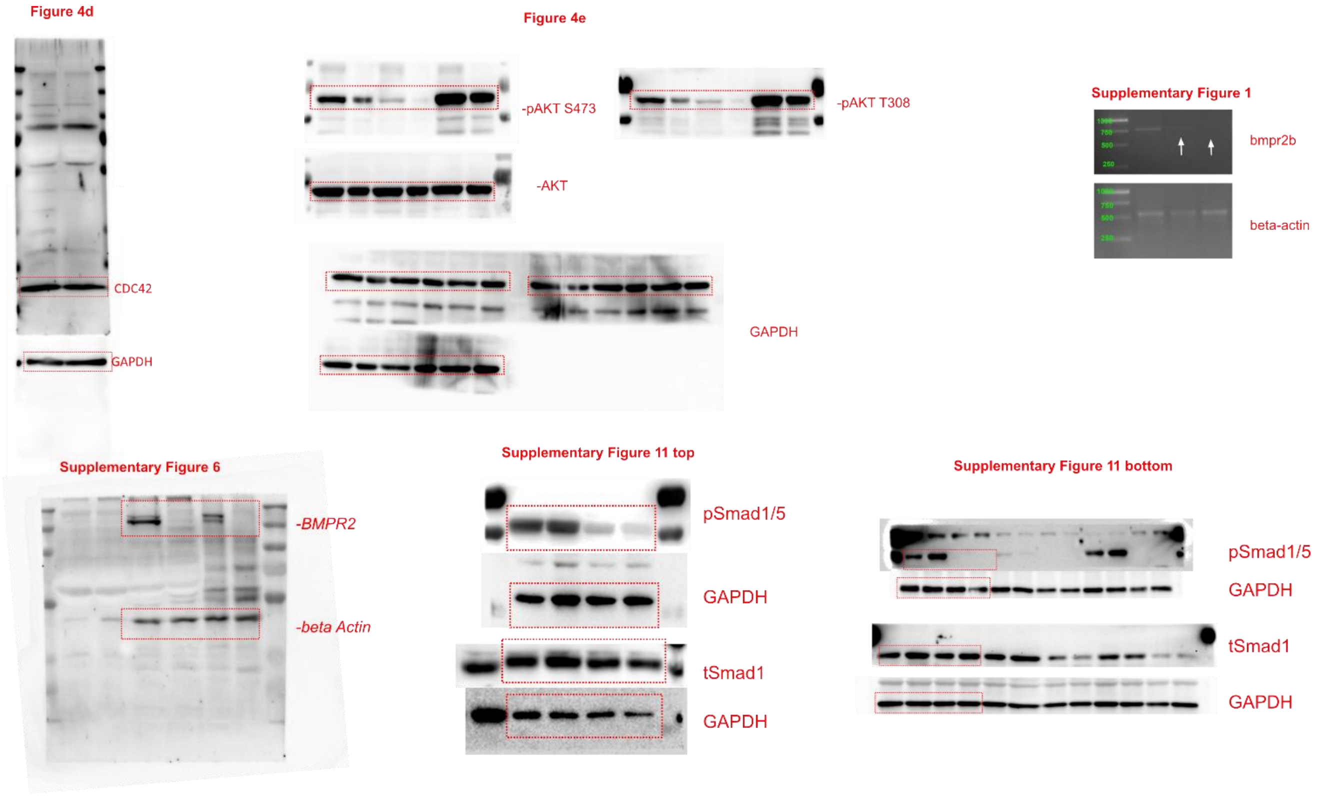

Supplementary Figure 26: Uncropped and unedited blot/gel images.
