# Supplementary material for: Endothelial tip-cell position, filopodia formation and biomechanics require BMPR2 expression and signaling
Source: Commun Biol. 2025 Jan 8;8:21. doi: 10.1038/s42003-024-07431-8 (PMC11711618; doi:10.1038/s42003-024-07431-8)
Supplement: Supplementary file 2 — Description of Additional Supplementary File [file 42003_2024_7431_MOESM2_ESM.pdf]

Description of additional supplementary data

File name: Supplementary Movie 1:
Description: Relaxation of BMP6- induced endothelial filopodia upon Rock inhibition.
HUVECs were transfected with BMPR2-GFP (white signal) and filopodia formation and stalling was imaged over 5 min. Left frame shows the first 2,5 min of filopodia extension and stabilization. After 2,5 min, 5µm Y-27632 dihydrochloride (ROCK inhibitor) was added on-line to the cells (right frame). The stalling in filopodia motility and partial retraction for some of them is visible on the right frame.

File name: Supplementary Movie 2:
Description: Life cell gap closure by BMPR2 wt (green) and BMPR2 deficient (magenta) ECs reveals BMPR2 requirement for different cell migration behavior**.**
ECs with corresponding genotype were seeded either in separate compartments of the gap insert (left side) or in equal numbers as mixture into both compartments similarly (right frame). Upon adherence, gap- forming insert was removed and cells were allowed to migrate for 16 hrs into the cell-free gap in the presence of EC activating medium. Left frame depicts on a more collective EC migration phenotype of BMPR2 deficient cells (magenta/ upper) over a rather disperse single cell-like migration process by BMPR2 expressing cells (green/ lower). Right frame shows that when competing for the gap closure process, BMPR2 expressing cells (green) outcompete BMPR2 deficient cells (magenta) during the gap closure process, indicating more sustained velocity in forward movement when BMPR2 is expressed.

File name: Supplementary Movie 3:
Description: Cytochalasin D and Y-27632 treatment both induce EC relaxation in Fibrin gels.
$BMPR2^{wt}$ ECs were coated onto Cytodex 3 microcarrier beads and embedded in a 3D fibrin gel allowing for sprouting angiogenesis under full medium condition. After selection and sprouting of first tip cells, sprout relaxation was induced by treatment with either 10 µM cytochalasin D (left) or 10µM Y-27632 (right). Video show similar sprouts relaxation for 60 minutes upon both treatments.

File name: Supplementary Movie 4
Description: Sprouting of BMPR2 wt and BMPR2 deficient 3D spheroids in fibrin gels.
$BMPR2^{wt}$ ECs or $BMPR2^{+/-}$ ECs were respectively coated onto Cytodex 3 microcarrier beads and embedded in a 3D fibrin gel allowing for sprouting angiogenesis under full medium condition. Spheroids were imaged every 6 hrs for a duration of 64 hrs. The resulting time-laps is presented

here and shows that after 64 hrs, $BMPR2^{wt}$ spheroids cover a larger sprouting area than $BMPR2^{+/-}$ spheroids.

File name: Supplementary Movie 5:
Description: Mosaic approach for BMPR2 wt (green) and BMPR2 deficient (magenta) spheroid assay in fibrin
Briefly $BMPR2^{wt}$ ECs (green) or $BMPR2^{+/-}$ ECs (magenta) were labeled separately before being coated together in a 1:1 ratio onto Cytodex 3 microcarrier beads. Resulting mosaic spheroids were embedded in a 3D fibrin gel allowing for sprouting angiogenesis under full medium condition and were imaged every 6 hrs for a duration of 64 hrs. The resulting time laps are presented here and show that $BMPR2^{wt}$ ECs outcompete $BMPR2^{+/-}$ ECs for the tip cell position during sprouting.

File name: Supplementary Data:
Description: Raw Sanger-sequencing data of PCR products sequencing from Supplementary Figure 2
