# Supplementary material for: Endothelial tip-cell position, filopodia formation and biomechanics require BMPR2 expression and signaling
Source: Commun Biol. 2025 Jan 8;8:21. doi: 10.1038/s42003-024-07431-8 (PMC11711618; doi:10.1038/s42003-024-07431-8)
Supplement: Supplementary file 9 — Reporting Summary [file 42003_2024_7431_MOESM9_ESM.pdf]

nature portfolio

Corresponding author(s): Petra Knaus

Last updated by author(s): Oct 31, 2024

Reporting Summary

Nature Portfolio wishes to improve the reproducibility of the work that we publish. This form provides structure for consistency and transparency in reporting. For further information on Nature Portfolio policies, see our Editorial Policies and the Editorial Policy Checklist.

Statistics

For all statistical analyses, confirm that the following items are present in the figure legend, table legend, main text, or Methods section.

n/a	Confirmed	
☒	☐	The exact sample size (*n*) for each experimental group/condition, given as a discrete number and unit of measurement
☐	☒	A statement on whether measurements were taken from distinct samples or whether the same sample was measured repeatedly
☐	☒	The statistical test(s) used AND whether they are one- or two-sided<br>*Only common tests should be described solely by name; describe more complex techniques in the Methods section.*
☒	☐	A description of all covariates tested
☐	☒	A description of any assumptions or corrections, such as tests of normality and adjustment for multiple comparisons
☐	☒	A full description of the statistical parameters including central tendency (e.g. means) or other basic estimates (e.g. regression coefficient) AND variation (e.g. standard deviation) or associated estimates of uncertainty (e.g. confidence intervals)
☒	☐	For null hypothesis testing, the test statistic (e.g. *F*, *t*, *r*) with confidence intervals, effect sizes, degrees of freedom and *P* value noted<br>*Give P values as exact values whenever suitable.*
☒	☐	For Bayesian analysis, information on the choice of priors and Markov chain Monte Carlo settings
☒	☐	For hierarchical and complex designs, identification of the appropriate level for tests and full reporting of outcomes
☒	☐	Estimates of effect sizes (e.g. Cohen's *d*, Pearson's *r*), indicating how they were calculated

Our web collection on statistics for biologists contains articles on many of the points above.

Software and code

Policy information about availability of computer code

Data collection	ZEN Blue 2.5, ZEN Black 2.5, Vilber FUSION FX
Data analysis	Graphpad Prism 9.3, FIJI, MATLAB R2021a, R Studio, Filoquant (ImageJ Plugin)

For manuscripts utilizing custom algorithms or software that are central to the research but not yet described in published literature, software must be made available to editors and reviewers. We strongly encourage code deposition in a community repository (e.g. GitHub). See the Nature Portfolio guidelines for submitting code & software for further information.

Data

Policy information about availability of data

All manuscripts must include a data availability statement. This statement should provide the following information, where applicable:

- Accession codes, unique identifiers, or web links for publicly available datasets
- A description of any restrictions on data availability
- For clinical datasets or third party data, please ensure that the statement adheres to our policy

The data supporting the findings of that study are available from the corresponding author upon request.

March 2021

Human research participants

Policy information about studies involving human research participants and Sex and Gender in Research.

Reporting on sex and gender	na
Population characteristics	na
Recruitment	na
Ethics oversight	na

Note that full information on the approval of the study protocol must also be provided in the manuscript.

Field-specific reporting

Please select the one below that is the best fit for your research. If you are not sure, read the appropriate sections before making your selection.

☒ Life sciences ☐ Behavioural & social sciences ☐ Ecological, evolutionary & environmental sciences

For a reference copy of the document with all sections, see nature.com/documents/nr-reporting-summary-flat.pdf

Life sciences study design

All studies must disclose on these points even when the disclosure is negative.

Sample size	For zebrafish data, each point correspond to an individual embryo.For filopodia quantification and migration assays, each point correspond to a single image. For 2D in vitro assays quantifications, each quantification point correspond to an individual cell. For 3D sprouting assays quantification each quantification point correspond to an individual spheroid or sprout as indicated in figure legend. For CDC42 relocation biosensor assay, each point correspond to a single cell.
Data exclusions	From Sprouting kinetic assay, were exluded quantification data from spheroids showing cell detachment from the beads, creating artfeacts of quantification that do not correspond to proper endothelial sprouting
Replication	All data sets correspond to at least ≥ 3 biological replicates. All attempts of replication were successful
Randomization	Randomization could not be performed due to necessary selection of specific field of views for filopodia and sprouting quantification.
Blinding	Blinding was not possible as most data were aqcuired via microscopy.

Reporting for specific materials, systems and methods

We require information from authors about some types of materials, experimental systems and methods used in many studies. Here, indicate whether each material, system or method listed is relevant to your study. If you are not sure if a list item applies to your research, read the appropriate section before selecting a response.

Materials & experimental systems

n/a	Involved in the study	
☐	☒	Antibodies
☐	☒	Eukaryotic cell lines
☒	☐	Palaeontology and archaeology
☐	☒	Animals and other organisms
☒	☐	Clinical data
☒	☐	Dual use research of concern

Methods

n/a	Involved in the study	
☒	☐	ChIP-seq
☒	☐	Flow cytometry
☒	☐	MRI-based neuroimaging

Antibodies

Antibodies used

Anti-phospho SMAD1/5 (Ser463/465) (41D10)	Cell Signaling Technology	#9516
anti-phospho-Akt (Ser473)	Cell Signaling Technology	#9271
anti-phospho MLC2 (Thr18/Ser19)	Cell Signaling Technology	#3674
anti-GAPDH	Cell Signaling Technology	#14C10

anti-Myc	Cell Signaling Technology	#9B11
anti-GFP	Cell Signaling Technology	#2956
anti-HA (HA-7)	Sigma-Aldrich	
anti- vinculin	Sigma-Aldrich	#V9131
anti-BMPR2	BD Bioscience	#612292
anti-CDC42	Abcam	#155940
anti- Beta-Catenin	BD Bioscience	#610153
anti-CD34	Abcam	#81289
Anti-Mouse Alexa Fluor 488 (goat)	Thermofisher Scientifics	A10684
Anti-Mouse Alexa Fluor 594 (goat)	Thermofisher Scientifics	A11020
Anti-Rabbit Alexa Fluor 488 (chicken)	Thermofisher Scientifics	A21441
Anti-Rabbit Alexa Fluor 594 (goat)	Thermofisher Scientifics	A11072
Goat anti rabbit 2ndary HRP-linked antibody	Dianova	111-035-144
Goat anti mouse 2ndary HRP-linked antibody	Dianova	115-035-068

Validation

All antibodies were validated by their respective manufacturers before use in this study.

Eukaryotic cell lines

Policy information about cell lines and Sex and Gender in Research

Cell line source(s)

Human EAhy926 BMPR2wt; WT ECs; WT (53, DOI: 10.1371/journal.pbio.3000557)

Human EAhy926 BMPR2 KO1 BMPR2+/-; BMPR2 deficient ECs; ECs with BMPR2 deficiency (53, DOI: 10.1371/journal.pbio.3000557)

Human umbilical vein endothelial cells (female) HUVEC (111, DOI: 10.1016/j.atherosclerosis.2019.10.007)

African green monkey kidney fibroblasts (SV40 T antigen) COS7 (from ATCC)

Authentication

None of the cell lines were authenticated

Mycoplasma contamination

All cells were tested negative for mycoplasma contamination

Commonly misidentified lines
(See ICLAC register)

No commonly misidentified cell lines were used for this study

Animals and other research organisms

Policy information about studies involving animals; ARRIVE guidelines recommended for reporting animal research, and Sex and Gender in Research

Laboratory animals

Zebrafish Tg(kdrl:EGFP) s843 (ZFIN identifier:ZDB-ALT-050916-14)

Wild animals

Provide details on animals observed in or captured in the field; report species and age where possible. Describe how animals were caught and transported and what happened to captive animals after the study (if killed, explain why and describe method; if released, say where and when) OR state that the study did not involve wild animals.

Reporting on sex

No Sex-based analysis were performed due to lack of relevance for this study

Field-collected samples

For laboratory work with field-collected samples, describe all relevant parameters such as housing, maintenance, temperature, photoperiod and end-of-experiment protocol OR state that the study did not involve samples collected from the field.

Ethics oversight

Handling of zebrafish was done in compliance with German and Brandenburg state law, carefully monitored by the local authority for animal protection (LUVG, Brandenburg, Germany; Animal protocol #2347-18-2015

Note that full information on the approval of the study protocol must also be provided in the manuscript.
